# Physicians’ Perceptions of and Satisfaction With Artificial Intelligence in Cancer Treatment: A Clinical Decision Support System Experience and Implications for Low-Middle–Income Countries

**DOI:** 10.2196/31461

**Published:** 2022-04-07

**Authors:** Srinivas Emani, Angela Rui, Hermano Alexandre Lima Rocha, Rubina F Rizvi, Sergio Ferreira Juaçaba, Gretchen Purcell Jackson, David W Bates

**Affiliations:** 1 Division of General Internal Medicine and Primary Care Brigham and Women's Hospital Harvard Medical School Boston, MA United States; 2 Department of Behavioral, Social, and Health Education Sciences Emory University Atlanta, GA United States; 3 Department of Community Health Federal University of Cearrá Fortaleza, CE Brazil; 4 Instituto do Câncer do Ceará Fortaleza, CE Brazil; 5 IBM Watson Health IBM Cambridge, MA United States; 6 Rodolfo Teofilo College Fortaleza CE Brazil; 7 Intuitive Surgical Sunnyvale, CA United States; 8 Departments of Pediatric Surgery, Pediatrics, and Biomedical Informatics Vanderbilt University Medical Center Nashville, TN United States; 9 Department of Healthcare Policy and Management Harvard School of Public Health Boston, MA United States

**Keywords:** artificial intelligence, cancer, low-middle–income countries, physicians, perceptions, Watson for Oncology, implementation, local context

## Abstract

As technology continues to improve, health care systems have the opportunity to use a variety of innovative tools for decision-making, including artificial intelligence (AI) applications. However, there has been little research on the feasibility and efficacy of integrating AI systems into real-world clinical practice, especially from the perspectives of clinicians who use such tools. In this paper, we review physicians’ perceptions of and satisfaction with an AI tool, Watson for Oncology, which is used for the treatment of cancer. Watson for Oncology has been implemented in several different settings, including Brazil, China, India, South Korea, and Mexico. By focusing on the implementation of an AI-based clinical decision support system for oncology, we aim to demonstrate how AI can be both beneficial and challenging for cancer management globally and particularly for low-middle–income countries. By doing so, we hope to highlight the need for additional research on user experience and the unique social, cultural, and political barriers to the successful implementation of AI in low-middle–income countries for cancer care.

## Introduction

The last several decades have witnessed the rapid growth of artificial intelligence (AI) applications in health care. AI is considered to comprise areas like machine learning, natural language processing, expert systems, and image and signal processing [1]. One group, who cited a study from Global Market Insights, noted that the use of AI in health care was expected to grow annually from 2016 to 2024, with expenditures increasing from US $760 million in 2016 to over US $10 billion in 2024 [2]. In a 2020 study, Global Market Insights noted that the AI in the health care market exceeded US $4 billion in 2020 and would grow at a compound annual growth rate of 33.7% between 2021 and 2027, with an expenditure of US $34.5 billion in 2027 [3]. This market growth has been accompanied by both national initiatives for AI and the rapid growth of academic literature on the use of AI in health care. For example, in India, an “AI for All” policy was established along with NITI (National Institution for Transforming India) Aayog—a Government of India think tank for formulating a national strategy for AI [4]. A bibliometric analysis of the literature reported in the *Journal of Medical Internet Research* found a growth rate of 45.15% in publications from 2014 to 2019, with 70.67% of all publications occurring in the same period [5]. This analysis also found the following top five health problems in the publications (in order of frequency): cancer, depression, Alzheimer disease, heart failure, and diabetes. Another review of AI applications in health care found the following areas of focus in the applications: sepsis, breast cancer, diabetic retinopathy, and polyps and adenomas [6]. Additionally, this review noted that the implementation of AI applications in real-world clinical settings is not widespread. Another recent review with a focus on patient safety outcomes also noted the lack of AI applications in real-world settings [7]. These articles, and others in the *Journal of Medical Internet Research* and elsewhere, have started to capture the use and role of AI in health care [8-11].

In this viewpoint, we contribute to this growing literature by detailing physicians’ experiences with an AI application—Watson for Oncology (WfO)—in the treatment of cancer. Physicians’ experiences with WfO are especially relevant, as the application has been implemented in diverse, real-world social and cultural settings. Our summary of physicians’ experiences with WfO relies on the extensive, published literature on this topic. After we describe physicians’ experiences with WfO, we comment about the opportunities and challenges associated with using AI for cancer care in low-middle–income countries (LMICs).

## The WfO Clinical Decision Support System Tool

WfO is a therapeutic oncology clinical decision support system (CDSS) that was trained by experts from the Memorial Sloan Kettering Cancer Center [12]. WfO uses both natural language processing and machine learning to process structured and unstructured data about patients with cancer and generate therapeutic options based on available evidence [13]. WfO provides 3 categories of therapeutic options: “recommended” treatments are those that adhere to the preferred training approach of the Memorial Sloan Kettering Cancer Center, treatments “for consideration” refer to alternative treatments based on evidence, and “not recommended” treatments refer to those that are not appropriate for certain patients [14]. Many early adopters of WfO measured the degree to which WfO therapeutic options were concordant with either clinical practice or the decisions of a multidisciplinary tumor board. WfO concordance rates varied widely across countries for many reasons, including differences in standard treatment guidelines, resource availability, and physician or patient preferences [15]. It is well recognized that concordance studies do not measure system accuracy but instead assess agreement with decisions made in practice, which may or may not reflect evidence-based decisions [16].

In this viewpoint, we focus on physicians’ perceptions of and satisfaction with WfO. We believe that an evaluation of physicians’ perceptions of this AI tool will provide valuable insights for the successful implementation of AI-based CDSSs for cancer treatment, especially in LMICs. Additionally, little is known about how physicians perceive the use of AI tools for cancer treatment. We present physicians’ perceptions of the advantages of, as well as the disadvantages and concerns with, AI in a real-world setting. Our summary relies on published literature on physicians’ perceptions of WfO implementation in a number of countries, including China, India, Mexico, South Korea, and Thailand. Multimedia Appendix 1 provides a comprehensive list of the studies on WfO [13-74].

## Advantages

The positive perceptions of WfO relate to the system’s ability to aid clinicians during the therapeutic decision-making process by quickly providing relevant scientific evidence. In China, a satisfaction survey, which was completed by 51 oncologists who used WfO, found that 86.3% of oncologists approved the quality of WfO and 88.2% approved the comprehensibility of WfO’s treatment options, justifications, and external literature [17]. The clinicians rated WfO highly in terms of its ability to provide evidence-based medicine medical education (score: 8.1/10) and literature assistance (score: 7.7/10), assist in medical care quality control (score: 7.3/10), act as a second-opinion consultation resource (score: 7.0/10), perform case reviews with a tumor board (score: 6.9/10), and provide decision support (score: 6.4/10). Overall, the oncologists recommended using WfO as a CDSS to other clinicians (score: 7.3/10). At Shanghai Tenth People’s Hospital, the multiple disciplinary team (MDT) also used WfO and found that their treatment plans became “more standardized, reasonable, and personalized” [18].

WfO’s ability to compare treatment options was tested in Mexico, where it was used for a total of 100 patient cases involving lung, breast, gastric, colon, and rectal cancers diagnosed within the last 5 years [19]. In terms of perceived utility, oncologists found WfO to be “very useful” in comparing treatment options. They reported that WfO might be especially valuable for individuals, such as medical students and residents who lack oncology experience, as well as clinics that do not have enough subspecialists. Several implementations of WfO in China indicate the role of WfO in enhancing the learning experience and efficiency of physicians, particularly junior physicians, and the facilitation of better diagnoses and treatment recommendations [20,21]. This perspective was also substantiated by students from Taipei Medical University Hospital in Taiwan who had limited clinical experience; by using WfO, they performed better on their colon cancer learning assessment than their peers who used traditional search methods and were more clinically experienced [22]. The study also found that students with less clinical experience felt that WfO was “clearer and more understandable” than information found through traditional methods.

WfO’s links to recent and relevant scientific information may provide treatment information that clinicians may not know. In India, an MDT changed their treatment recommendations for 136 of 1000 cases of breast, lung, colon, and rectal cancers because of the data provided by WfO [23]. For 55% of those cases, WfO provided recent evidence of newer treatments. For 30% of the cases, WfO provided new information about genotypic and phenotypic data. For 15% of the cases, WfO provided information on evolving clinical experiences, which influenced the MDT to change their treatment decisions. These results demonstrate the potential of WfO to positively impact cancer outcomes by providing scientific evidence and up-to-date information on clinical guidelines. In a separate study that focused on adjuvant systemic therapy for breast carcinoma, treatment decisions were changed for 4 of 11 patients after the MDT reviewed WfO’s recommendations and EndoPredict (Myriad Genetics Inc) test reports [24]. WfO was able to aid clinicians in providing personalized cancer care while addressing the difficulties of staying informed on evolving cancer guidelines and studies.

Another aspect that must be considered is whether WfO can be useful as a CDSS. At the Instituto Câncer do Ceará in Brazil, a majority of oncologists chose the “agree” or “strongly agree” option for statements that were used to confirm if WfO meets the “CDS Five Rights” criteria [25]. The “CDS Five Rights” contain clinical quality criteria for determining if a CDSS offers benefits that are optimal for a given setting [75]. In the study, 6 of the 7 oncologists at the Instituto Câncer do Ceará believed that WfO provided relevant information that resulted in action being taken and presented the information in a manner that positively aligned with their individual workflows. Further, 5 oncologists agreed that the additional details for each treatment option were easily comprehensible, and 4 oncologists agreed that WfO exceeded their expectations as a CDSS tool for patient management.

## Disadvantages and Concerns

Although WfO appears to be useful for displaying information in a succinct and timely manner, there are concerns regarding the system’s usability and integration into clinician workflows. First, at sites without integrated patient record systems, some users found manual data entry to be a burdensome process [13,26]. At Manipal Hospital in India, it was observed that acclimation to the system reduced the time needed for each patient case [27]. The mean time needed to collect and enter data for nonmetastatic diseases was 20 minutes. This was reduced to 12 minutes after an increased acquaintance of 10 cases with WfO. In comparison, the time needed to collect and enter data for metastatic diseases was 5 to 7 minutes longer than that for localized diseases. On average, WfO took a median of 40 seconds to capture, analyze, and provide treatment recommendations. For physicians with a high patient load, the time needed to enter information into the system may be an issue. Users also want WfO to provide an explanation of its process for scoring and ranking treatment options [26]. In doing so, users would feel more comfortable with trusting the information and recommendations provided by WfO.

A second important concern that has been identified in studies is localizing WfO’s treatment recommendations to the country of implementation. In the previously mentioned satisfaction study conducted in China, 66.7% of physicians recommended that WfO should integrate data on locally available treatments to improve the system [17]. For example, WfO did not take into consideration whether the immunotherapy drugs it recommended had been approved by the China Food and Drug Administration. Physicians also chose chemotherapy instead of WfO’s recommended medication because the medication was too expensive for patients. Similar challenges were found for WfO users in Mexico and Thailand [19,28]. In Mexico, clinicians deviated from WfO’s recommendations due to the high costs associated with them and the fact that they did not adhere to Mexican cancer treatment guidelines [19]. In Thailand, oncologists preferred basing their treatment recommendations on other countries’ guidelines instead of US guidelines [28].

## Implications for LMICs

In 2012, 65% of all cancer deaths worldwide occurred in LMICs, and the projection for 2030 is that this will increase to 75% [76]. LMICs may also be experiencing an even higher burden from cancer than that experienced by high-income countries (HICs) for several reasons. LMICs have restrained funding and often lack optimal cancer registries and surveillance data; thus, they are unable to implement evidence-based cancer control programs [76]. Treatment modalities are also more limited in LMICs than in HICs; radiotherapy and chemotherapy are available in 43% to 51% of LMICs but are available in 94% of HICs [77]. However, there is a high demand for such therapies, as 5 million new people annually are estimated to need radiation therapy in LMICs [78]. LMICs also lack specialized medical personnel, such as oncologists and oncology nurses, who are needed to address those affected by cancer in LMICs [79]. According to a World Health Organization report, LMICs have the lowest density of health care workers in comparison to HICs, where the density of health care workers is significantly higher [80]. A lack of health care workers for serving the population makes offering high-quality, personalized care a difficult task.

Oncologists also often require the expertise of their colleagues and additional literary resources to determine a course of treatment for unique cancer cases. Gaining access to high-quality medical information is key for creating an appropriate treatment plan, but oncologists may need additional help with sorting information that is both relevant to their patients and viable in terms of what resources are available. AI-based platforms such as WfO may be able to address the growing challenges of providing cancer treatment plans in LMICs. AI can address issues of access to knowledge bases in a comprehensive and easy-to-access manner. The ability of AI tools to quickly provide evidence-based cancer treatment options would be especially helpful in low-resource settings where the lack of time, expertise, and other needed resources can become a barrier to providing care. Using AI in this manner may also promote international partnerships on cancer therapy research and standardize guidelines for certain cancer types. The studies reviewed in this viewpoint demonstrate the potential of AI to reduce the cognitive burdens of less experienced physicians who would benefit from additional medical education resources.

The experiences with WfO in different settings also reveal a positive perception of AI with regard to its ability to reassure clinicians and confirm their interpretations of data and the potential of such a tool to do so in an LMIC. The ability of AI to act as a second opinion resource and standardize treatments may prove especially useful for cancer care in LMICs where the likelihood of receiving comprehensive care and achieving positive outcomes is lower than that in HICs. A lack of available specialized medical personnel in LMICs, especially in rural regions, is one of the factors contributing to poor cancer outcomes in LMICs [81]. The ability of AI tools, such as WfO, to provide subspecialty treatment information makes such tools a much-needed resource that existing physicians can use to meet population demands, especially in rural areas, as envisioned for the use of AI in an LMIC like India [4,82]. Approximately 60 oncologists serve over 300 million people in West Africa, and only 2000 oncologists are available for 10 million patients in India [83-85]. WfO’s open-access information, which can be used to supplement self-paced learning, would be an ideal resource for cancer physicians in LMICs where medical education resources are lacking [86,87].

The use of an AI tool such as WfO in LMICs also poses certain challenges. The technological challenges that are unique to LMICs and should be mentioned include access to the internet, technology training, and whether local technology teams would be able to address technical issues [87]. Providing a decision support tool, such as WfO, that is user-friendly and aligns with daily workflows is essential for implementation in LMICs, where physicians’ experiences with technology can vary [87]. Additionally, there is concern about whether AI tools would exacerbate the divide in health care access and use, especially with respect to socioeconomic status. There is a fear that AI would recommend treatment options that patients cannot afford or that only high-income and educated patients who are aware of AI tools may benefit from the use of AI in LMICs [4,19,28] Another important concern—one that applies to a tool such as WfO—is that the data and training of AI tools may not incorporate patient characteristics into treatment recommendations. For example, in China, local patient characteristics such as gene mutations and the weaker physiques of Chinese patients, which can influence treatment recommendations, were not accounted for in WfO recommendations [20]. Similarly, the need to consider the presence of multiple ethnic groups in countries like India during the implementation of AI tools developed by Western countries will be an important factor to address in LMICs [88].

## Conclusion

It is undeniable that oncology physicians in LMICs need as much additional support as possible. The implementation of AI tools, such as WfO, in different settings has revealed that access to a second opinion CDSS resource, concise scientific evidence, and international clinical guidelines can help physicians feel more confident in their final treatment decisions. To improve the clinical utility of AI tools such as WfO, it is necessary that the experiences and satisfaction of physicians who use such tools are explored more in-depth, especially those of physicians in LMICs. These perspectives are especially key to tailoring AI systems for use in real-world clinical settings [6,7]. Such perspectives are of course embedded in the local social, cultural, and political LMIC contexts within which AI is implemented and the ways in which local contexts can shape the use of AI. We are gaining experience with respect to the implementation of AI tools, such as WfO, in real-world settings for the treatment of cancer. However, we still need to address some of the challenges in the “last mile” stage of implementation, specifically those related to local contexts [89].

## Appendix

Multimedia Appendix 1 Studies on Watson for Oncology (WfO).

## Abbreviations

AI: artificial intelligence
CDSS: clinical decision support system
HIC: high-income country
LMIC: low-middle–income country
MDT: multiple disciplinary team
NITI: National Institution for Transforming India
WfO: Watson for Oncology
