# Supplementary material for: Physicians’ Perceptions of and Satisfaction With Artificial Intelligence in Cancer Treatment: A Clinical Decision Support System Experience and Implications for Low-Middle–Income Countries
Source: JMIR Cancer. 2022 Apr 7;8(2):e31461. doi: 10.2196/31461 (PMC9030908; doi:10.2196/31461)
Supplement: Multimedia Appendix 1 [file cancer_v8i2e31461_app1.docx]

**Appendix 1. Studies on Watson for Oncology (WfO)**

**Note**: The references listed in the appendix are arranged chronologically. These references correspond to references 13-74 in the main article.

1. Bach P, Zauderer MG, Gucalp A, Epstein AS, Norton L, Seidman AD, et al. Beyond Jeopardy!: Harnessing IBM's Watson to improve oncology decision making. J Clin Oncol 2013 May 20;31(15_suppl):6508. [doi: 10.1200/jco.2013.31.15_suppl.6508]

2. Epstein AS, Zauderer MG, Gucalp A, Seidman AD, Caroline A, Fu J, et al. Next steps for IBM Watson Oncology: Scalability to additional malignancies. J Clin Oncol 2014 May 20;32(15_suppl):6618. [doi: 10.1200/jco.2014.32.15_suppl.6618]

3. Zauderer MG, Gucalp A, Epstein AS, Seidman AD, Caroline A, Granovsky S, et al. Piloting IBM Watson Oncology within Memorial Sloan Kettering’s regional network. J Clin Oncol 2014 May 20;32(15_suppl):e17653. [doi: 10.1200/jco.2014.32.15_suppl.e17653]

4. Fu J, Gucalp A, Zauderer MG, Epstein AS, Kris MG, Keesing J, et al. Steps in developing Watson for Oncology, a decision support system to assist physicians choosing first-line metastatic breast cancer (MBC) therapies: Improved performance with machine learning. J Clin Oncol 2015 May 20;33(15_suppl):566. [doi: 10.1200/jco.2015.33.15_suppl.566]

5. Kris MG, Gucalp A, Epstein AS, Seidman AD, Fu J, Keesing J, et al. Assessing the performance of Watson for oncology, a decision support system, using actual contemporary clinical cases. J Clin Oncol 2015 May 20;33(15_suppl):8023. [doi: 10.1200/jco.2015.33.15_suppl.8023]

6. Seidman AD, Pilewskie ML, Robson ME, Kelvin JF, Zauderer MG, Epstein AS, et al. Integration of multi-modality treatment planning for early stage breast cancer (BC) into Watson for Oncology, a Decision Support System: Seeing the forest and the trees. J Clin Oncol 2015 May 20;33(15_suppl):e12042. [doi: 10.1200/jco.2015.33.15_suppl.e12042]

7. Herath DH, Wilson-Ing D, Ramos E, Morstyn G. Assessing the natural language processing capabilities of IBM Watson for oncology using real Australian lung cancer cases. J Clin Oncol 2016 May 20;34(15_suppl):e18229. [doi: 10.1200/JCO.2016.34.15_suppl.e18229]

8. Somashekhar SSP, Kumar R, Kumar A, Patil P, Rauthan A. 551PD Validation study to assess performance of IBM cognitive computing system Watson for oncology with Manipal multidisciplinary tumour board for 1000 consecutive cases: An Indian experience. Ann Oncol 2016 Dec;27:ix179. [doi: 10.1016/S0923-7534(21)00709-2]

9. Suwanvecho S, Suwanrusme H, Sangtian M, Norden AD, Urman A, Hicks A, et al. Concordance assessment of a cognitive computing system in Thailand. J Clin Oncol 2017 May 30;35(15_suppl):6589. [doi: 10.1200/JCO.2017.35.15_suppl.6589]

10. Somashekhar S, Sepúlveda M, Norden AD, Rauthan A, Arun K, Patil P, et al. Early experience with IBM Watson for Oncology (WFO) cognitive computing system for lung and colorectal cancer treatment. J Clin Oncol 2017 May 30;35(15_suppl):8527. [doi: 10.1200/JCO.2017.35.15_suppl.8527]

11. Sarre-Lazcano C, Alonso AA, Melendez FDH, Arrieta O, Norden AD, Urman A, et al. Cognitive computing in oncology: A qualitative assessment of IBM Watson for Oncology in Mexico. J Clin Oncol 2017 May 30;35(15_suppl):e18166. [doi: 10.1200/jco.2017.35.15_suppl.e18166]

12. Baek JH, Ahn SM, Urman A, Kim YS, Ahn HK, Won PS, et al. Use of a cognitive computing system for treatment of colon and gastric cancer in South Korea. J Clin Oncol 2017 May 30;35(15_suppl):e18204. [doi: 10.1200/JCO.2017.35.15_suppl.e18204]

13. Lim S, Lee KB. Use of a cognitive computing system for treatment of cervical cancer. J Gynecol Oncol 2017 Sep;28(5):e67 [FREE Full text] [doi: 10.3802/jgo.2017.28.e67] [Medline: 28657228]

14. Zhou N, Lv H, Zhang C, Li T, Zhu J, Jiang M, et al. P1.01-069 Clinical experience with IBM Watson for Oncology (WFO) cognitive system for lung cancer treatment in China. J Thorac Oncol 2017 Nov;12(11):S1921. [doi: 10.1016/j.jtho.2017.09.723]

15. Yue L, Yang L. Clinical experience with IBM Watson for Oncology (WFO) for multiple types of cancer patients in China. Ann Oncol 2017 Nov;28:x162. [doi: 10.1093/annonc/mdx676.024]

16. Zhang XC, Zhou N, Zhang CT, Lv HY, Li TJ, Zhu JJ, et al. Concordance study between IBM Watson for Oncology (WFO) and clinical practice for breast and lung cancer patients in China. Ann Oncol 2017 Nov 01;28(Supplement 10):x170. [doi: 10.1093/annonc/mdx678.001]

17. Somashekhar SP, Kumarc R, Rauthan A, Arun KR, Ramya YE. Abstract S6-07: Double blinded validation study to assess performance of IBM artificial intelligence platform, Watson for oncology in comparison with Manipal multidisciplinary tumour board – First study of 638 breast cancer cases. Cancer Res 2017;77(4_Supplement):S6-07. [doi: 10.1158/1538-7445.sabcs16-s6-07]

18. Somashekhar SP, Sepúlveda MJ, Puglielli S, Norden AD, Shortliffe EH, Rohit Kumar C, et al. Watson for Oncology and breast cancer treatment recommendations: agreement with an expert multidisciplinary tumor board. Ann Oncol 2018 Feb 01;29(2):418-423 [FREE Full text] [doi: 10.1093/annonc/mdx781] [Medline: 29324970]

19. Wu A, Chen PJ, Li TI, Dankwa-Mullan I, Sun TT, Rhee K. Real world survival outcomes in patients with high risk stage II colon cancer at a Beijing Cancer Hospital. J Clin Oncol 2018 May 20;36(15_suppl):e15670-e15670. [doi: 10.1200/JCO.2018.36.15_suppl.e15670]

20. Fang J, Zhu Z, Wang H, Hu F, Liu Z, Guo X, et al. The establishment of a new medical model for tumor treatment combined with Watson for Oncology, MDT and patient involvement. J Clin Oncol 2018 Jun 01;36(15_suppl):e18504. [doi: 10.1200/jco.2018.36.15_suppl.e18504]

21. Jiang Z, Xu F, Sepúlveda M, Li J, Wang H, Liu Z, et al. Concordance, decision impact and guidelines adherence using artificial intelligence in high-risk breast cancer. J Clin Oncol 2018 Jun 01;36(15_suppl):e18566. [doi: 10.1200/JCO.2018.36.15_suppl.e18566]

22. Yuwen D, Zhang W, Wu J, Zhang J, Shen Y, Shi J, et al. Concordance evaluation of an artificial intelligence technology with a multidisciplinary tumor board in gastric cancer. J Clin Oncol 2018 Jun 01;36(15_suppl):e18569. [doi: 10.1200/JCO.2018.36.15_suppl.e18569]

23. Chen PJ, Sun TT, Li TI, Dankwa-Mullan I, Urman A, Wang CK, et al. Can AI technology augment tumor board treatment decisions for stage II colon cancer care? J Clin Oncol 2018 Jun 01;36(15_suppl):e18582. [doi: 10.1200/JCO.2018.36.15_suppl.e18582]

24. Suwanrusme H, Issarachai S, Umsawasdi T, Suwanvecho S, Decha W, Dankwa-Mullan I, et al. Concordance assessment of a clinical decision support software in patients with solid tumors. J Clin Oncol 2018 Jun 01;36(15_suppl):e18584. [doi: 10.1200/JCO.2018.36.15_suppl.e18584]

25. Saiz FJS, Urman A, Sanders C, Britt MW, Nielsen R, Stevens RJ. IBM Watson Evidence Service (WES): A system for retrieval, summation and insight generation of relevant clinical evidence for personalized oncology. J Clin Oncol 2018 Jun 01;36(15_suppl):e18588. [doi: 10.1200/JCO.2018.36.15_suppl.e18588]

26. Kim YY, Oh SJ, Chun YS, Lee WK, Park HK. Gene expression assay and Watson for Oncology for optimization of treatment in ER-positive, HER2-negative breast cancer. PLoS One 2018 Jul 06;13(7):e0200100. [doi: 10.1371/journal.pone.0200100] [Medline: 29979736]

27. Keikes L, Medlock S, van de Berg DJ, Zhang S, Guicherit OR, Punt CJA, et al. The first steps in the evaluation of a "black-box" decision support tool: a protocol and feasibility study for the evaluation of Watson for Oncology. J Clin Transl Res 2018 Jul 27;3(Suppl 3):411-423 [FREE Full text] [Medline: 30873490]

28. Soochit A, Zhang C, Li T, Dankwa-Mullan I, Liu J. Concordance assessment of an artificial intelligence decision support tool for primary and recurrent cervical cancer at an academic cancer center. In: Int J Gynecol Cancer. 2018 Presented at: 17th Biennial Meeting of the International Gynecologic Cancer Society; September 14-16, 2018; Kyoto, Japan.

29. Zhang C, Soochit A, Dankwa-Mullan I, Li T, Liu J. Evaluation of personalized treatment with artificial intelligence decision-support tools for patients with ovarian cancer. In: Int J Gynecol Cancer. 2018 Presented at: 17th Biennial Meeting of the International Gynecologic Cancer Society; September 14-16, 2018; Kyoto, Japan.

30. Liu C, Liu X, Wu F, Xie M, Feng Y, Hu C. Using artificial intelligence (Watson for Oncology) for treatment recommendations amongst Chinese patients with lung cancer: Feasibility study. J Med Internet Res 2018 Sep 25;20(9):e11087 [FREE Full text] [doi: 10.2196/11087] [Medline: 30257820]

31. Graham DMA, McNamara DM, Waintraub SE, Goldberg SL, Norden AD, Hervey J, et al. Are treatment recommendations provided by cognitive computing supported by real world data (Watson for Oncology with Cota RWE) concordant with expert opinions? Ann Oncol 2018 Oct;29:viii571. [doi: 10.1093/annonc/mdy297.031]

32. Waintraub SE, McNamara DM, Graham DMA, Goldberg SL, Norden AD, Hervey J, et al. Can the cognitive computing system Watson for Oncology with Cota RWE help oncologists deliver subspecialist level care? 2018 Presented at: 36th Annual Chemotherapy Foundation Symposium (CFS); November 7-9, 2018; New York, New York.

33. Lee WS, Ahn SM, Chung JW, Kim KO, Kwon KA, Kim Y, et al. Assessing concordance with Watson for Oncology, a cognitive computing decision support system for colon cancer treatment in Korea. JCO Clin Cancer Inform 2018 Dec;2:1-8 [FREE Full text] [doi: 10.1200/CCI.17.00109] [Medline: 30652564]

34. Choi YI, Chung JW, Kim KO, Kwon KA, Kim YJ, Park DK, et al. Concordance rate between clinicians and Watson for Oncology among patients with advanced gastric cancer: Early, real-world experience in Korea. Can J Gastroenterol Hepatol 2019 Feb 03;2019:8072928 [FREE Full text] [doi: 10.1155/2019/8072928] [Medline: 30854352]

35. Waintraub SC, Pecora AL. Cognitive computing and real world data in a breast cancer clinic. 2019 Presented at: Machine Learning & AI for Healthcare: A HIMSS Event; Orlando, Florida; February 11, 2019 URL: https://365.himss.org/sites/ himss365/files/365/handouts/552672001/handout-MLAI09.pdf

36. Kim EJ, Woo HS, Cho JH, Sym SJ, Baek JH, Lee WS, et al. Early experience with Watson for oncology in Korean patients with colorectal cancer. PLoS One 2019 Mar 25;14(3):e0213640. [doi: 10.1371/journal.pone.0213640] [Medline: 30908530]

37. Hamilton JG, Garzon MG, Westerman JS, Shuk E, Hay JL, Walters C, et al. "A Tool, Not a Crutch": Patient perspectives about IBM Watson for Oncology trained by Memorial Sloan Kettering. J Oncol Pract 2019 Apr;15(4):e277-e288 [FREE Full text] [doi: 10.1200/JOP.18.00417] [Medline: 30689492]

38. Somashekhar SP, Sepúlveda MJ, Shortliffe EH, C RK, Rauthan A, Patil P, et al. A prospective blinded study of 1000 cases analyzing the role of artificial intelligence: Watson for oncology and change in decision making of a Multidisciplinary Tumor Board (MDT) from a tertiary care cancer center. J Clin Oncol 2019 May 26;37(15_suppl):6533. [doi: 10.1200/jco.2019.37.15_suppl.6533]

39. Li T, Chen C, Zhang SS, Dankwa-Mullan I, Chen A, Preininger A, et al. Deployment and integration of a cognitive technology in China: Experiences and lessons learned. J Clin Oncol 2019 May 26;37(15_suppl):6538. [doi: 10.1200/jco.2019.37.15_suppl.6538]

40. Liang J, Li T, Zhang SS, Chen C, VanHouten C, Preininger A, et al. Reasons for discordance in treatment approaches between oncology practice and clinical decision support in China. J Clin Oncol 2019 May 26;37(15_suppl):6555. [doi: 10.1200/jco.2019.37.15_suppl.6555]

41. Fang J, Guo X, Zhu Z, Wang H, Hu F, Chen J, et al. Watson for Oncology applied to teaching and remote consulting model. J Clin Oncol 2019 May 26;37(15_suppl):6545. [doi: 10.1200/JCO.2019.37.15_suppl.6545]

42. Suwanvecho S, Shortliffe EH, Suwanrusme H, Issarachai S, Jirakulaporn T, Taechakraichana N, et al. A blinded evaluation of a clinical decision-support system at a regional cancer care center. J Clin Oncol 2019 May 26;37(15_suppl):6553. [doi: 10.1200/JCO.2019.37.15_suppl.6553]

43. Saiz FJS, Sanders C, Stevens RJ, Nielsen R, Britt MW, Preininger A, et al. Use of machine learning to identify relevant research publications in clinical oncology. J Clin Oncol 2019 May 26;37(15_suppl):6558. [doi: 10.1200/JCO.2019.37.15_suppl.6558]

44. Chen CY, Hung HC, Chiu HY, Wei PL, Kuo PL, Chiou JF, et al. Enhancing evidence-based medicine skills in oncology training with cognitive technology. J Clin Oncol 2019 May 26;37(15_suppl):10532. [doi: 10.1200/jco.2019.37.15_suppl.10532]

45. Fang J, Guo X, Zhu Z, Wang H, Hu F, Chen J, et al. Quality control system of Watson for oncology: Artificial intelligence for supporting clinical decisions in oncology. J Clin Oncol 2019 May 26;37(15_suppl):6616. [doi: 10.1200/JCO.2019.37.15_suppl.6616]

46. Chen ZB, Chen SL, Liang RM, Peng ZW, Shen JX, Zhu WJ, et al. Can artificial intelligence support the clinical decision making for Barcelona clinic liver cancer stage 0/a hepatocellular carcinoma in China? J Clin Oncol 2019 May 26;37(15_suppl):e15634. [doi: 10.1200/JCO.2019.37.15_suppl.e15634]

47. Rocha HAL, Dankwa-Mullan I, Juacaba SF, Willis V, Arriaga YE, Jackson GP, et al. Shared-decision making in prostate cancer with clinical decision-support. J Clin Oncol 2019 May 26;37(15_suppl):e16576. [doi: 10.1200/JCO.2019.37.15_suppl.e16576]

48. Somashekhar SP, Yethadka R, C RK, Rajgopal AK, Rauthan A, Patil P. Triple blinded prospective study assessing the impact of genomics: EndoPredict and artificial intelligence Watson for Oncology (WFO) on MDT’s decision of adjuvant systemic therapy for hormone receptor positive early breast carcinoma. J Clin Oncol 2019 May 26;37(15_suppl):e18013. [doi: 10.1200/jco.2019.37.15_suppl.e18013]

49. Liang J, Dankwa-Mullan I, Ren Y, Chen A, Willis V, Jackson G, et al. Employing an oncology decision-support system to quantify treatment variation. J Clin Oncol 2019 May 26;37(15_suppl):e18067. [doi: 10.1200/JCO.2019.37.15_suppl.e18067]

50. Rocha HAL, Dankwa-Mullan I, Juacaba SF, Preininger A, Felix W, Thompson JV, et al. An evaluation of artificial intelligence-based clinical decision supports use in Brazil. J Clin Oncol 2019 May 26;37(15_suppl):e18081. [doi: 10.1200/jco.2019.37.15_suppl.e18081]

51. Wang Z, Yu Z, Zhang X. Artificial intelligence-based clinical decision-support system improves cancer treatment and patient satisfaction. J Clin Oncol 2019 May 26;37(15_suppl):e18303. [doi: 10.1200/JCO.2019.37.15_suppl.e18303]

52. Yu Z, Wang Z, Ren X, Lou D, Li X, Liu H, et al. Practical exploration and research of Watson for oncology clinical decision support system in real-world and localized practice. J Clin Oncol 2019 May 26;37(15_suppl):e18304. [doi: 10.1200/JCO.2019.37.15_suppl.e18304]

53. Liang J, Tang C, Wang X, Guo Z, Ni J, Xu W, et al. Impact of decision-support system and guideline treatment concordance on response rate in advanced lung cancer. J Clin Oncol 2019 May 26;37(15_suppl):e20006. [doi: 10.1200/JCO.2019.37.15_suppl.e20006]

54. Simon G, DiNardo CD, Takahashi K, Cascone T, Powers C, Stevens R, et al. Applying artificial intelligence to address the knowledge gaps in cancer care. Oncologist 2019 Jun;24(6):772-782 [FREE Full text] [doi: 10.1634/theoncologist.2018-0257] [Medline: 30446581]

55. Zhou N, Zhang CT, Lv HY, Hao CX, Li TJ, Zhu JJ, et al. Concordance study between IBM Watson for Oncology and clinical practice for patients with cancer in China. Oncologist 2019 Jun;24(6):812-819 [FREE Full text] [doi: 10.1634/theoncologist.2018-0255] [Medline: 30181315]

56. Kim D, Kim YY, Lee JH, Chung YS, Choi S, Kang JM, et al. A comparative study of Watson for Oncology and tumor boards in breast cancer treatment. Korean Journal of Clinical Oncology 2019 Jun 30;15(1):3-6 [FREE Full text] [doi: 10.14216/kjco.19002]

57. Kim M, Kim BH, Kim JM, Kim EH, Kim K, Pak K, et al. Concordance in postsurgical radioactive iodine therapy recommendations between Watson for Oncology and clinical practice in patients with differentiated thyroid carcinoma. Cancer 2019 Aug 15;125(16):2803-2809 [FREE Full text] [doi: 10.1002/cncr.32166] [Medline: 31216369]

58. Chen S, Chen Z, Xiao H, Peng Z, Peng S, Kuang M. IDDF2019-ABS-0095 Can artificial intelligence support the clinical decision making for hepatocellular carcinoma? Gut 2019;68:A134. [doi: 10.1136/gutjnl-2019-IDDFAbstracts.263]

59. Tian Y, Liu X, Wang Z, Cao S, Liu Z, Ji Q, et al. Concordance between Watson for Oncology and a multidisciplinary clinical decision-making team for gastric cancer and the prognostic implications: Retrospective study. J Med Internet Res 2020 Feb 20;22(2):e14122 [FREE Full text] [doi: 10.2196/14122] [Medline: 32130123]

60. Zou FW, Tang YF, Liu CY, Ma JA, Hu CH. Concordance study between IBM Watson for Oncology and real clinical practice for cervical cancer patients in China: A retrospective analysis. Front Genet 2020 Mar 24;11:200 [FREE Full text] [doi: 10.3389/fgene.2020.00200] [Medline: 32265980]

61. Saiz FS, Sanders C, Stevens R, Nielsen R, Britt M, Yuravlivker L, et al. Artificial intelligence clinical evidence engine for automatic identification, prioritization, and extraction of relevant clinical oncology research. JCO Clin Cancer Inform 2021 Jan;5:102-111 [FREE Full text] [doi: 10.1200/CCI.20.00087] [Medline: 33439724]

62. Suwanvecho S, Suwanrusme H, Jirakulaporn T, Issarachai S, Taechakraichana N, Lungchukiet P, et al. Comparison of an oncology clinical decision-support system's recommendations with actual treatment decisions. J Am Med Inform Assoc 2021 Mar 18;28(4):832-838 [FREE Full text] [doi: 10.1093/jamia/ocaa334] [Medline: 33517389]

**Note**: The references listed in the appendix are arranged chronologically. These references correspond to references 13-74 in the main article.
